# Asymmetrical Flow Field-Flow Fractionation on Virus and Virus-Like Particle Applications

**DOI:** 10.3390/microorganisms7110555

**Published:** 2019-11-12

**Authors:** Katri Eskelin, Minna M. Poranen, Hanna M. Oksanen

**Affiliations:** Molecular and Integrative Biosciences Research Programme, Faculty of Biological and Environmental Sciences, Viikinkaari 9, University of Helsinki, FI-00014 Helsinki, Finland; minna.poranen@helsinki.fi

**Keywords:** virus, virus-like particle, field-flow fractionation, size-based separation, macromolecular complex, virus purification, light scattering

## Abstract

Asymmetrical flow field-flow fractionation (AF4) separates sample components based on their sizes in the absence of a stationary phase. It is well suited for high molecular weight samples such as virus-sized particles. The AF4 experiment can potentially separate molecules within a broad size range (~10^3^−10^9^ Da; particle diameter from 2 nm to 0.5−1 μm). When coupled to light scattering detectors, it enables rapid assays on the size, size distribution, degradation, and aggregation of the studied particle populations. Thus, it can be used to study the quality of purified viruses and virus-like particles. In addition to being an advanced analytical characterization technique, AF4 can be used in a semi-preparative mode. Here, we summarize and provide examples on the steps that need optimization for obtaining good separation with the focus on virus-sized particles.

## 1. Short Introduction to Asymmetrical Flow Field-Flow Fractionation

**Figure 1:**
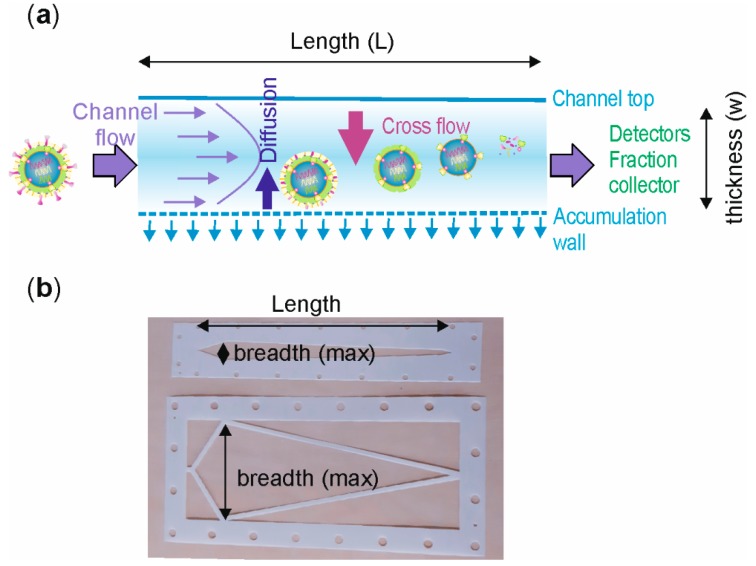
(**a**) Principle of asymmetrical flow field-flow fractionation (AF4). Cross flow forces the sample components towards the accumulation wall, which consists of an ultrafiltration membrane layered on top of a permeable ceramic frit through which the cross-flow is pumped out. Sample components diffuse against the cross-flow force depending on their diffusion coefficients that are size-dependent. Channel flow that moves through the channel has the highest velocity in the middle of the channel. It transports sample components towards the detectors. Small sample components reach the highest velocities and elute first. In practice, the sample components occupy ~1% of the total channel thickness (w) and the rest of the flow is sample free. Virus particles are not drawn to scale. (**b**) Channel shape, length (L), maximum breadth (b_max_), and nominal thickness (w, see **a**) are determined by the spacer. Due to the spacer design, the breadth of the channel decreases linearly towards the outlet end of the channel. Commercial spacers for standard analytical (top) and semi-preparative (bottom) AF4 channels from Postnova are shown.

In asymmetrical flow field-flow fractionation (AF4), sample components are separated in a trapezoid-shaped flat channel using the forces generated by two simultaneous flows, as follows: The channel flow (V_out_) and the cross flow (V_c_) (Figure 1a). The channel has a permeable bottom plate and an impermeable see-through top plate (Figure 1a), which are separated by a spacer with a typical width of 250−500 μm (Figure 1b). The porous bottom plate is layered with an ultrafiltration membrane of desired material and molecular-weight cut-off (MWCO), determining the lowest size limit for sample components that are retained in the channel. The membrane and the bottom plate form the accumulation wall. The laminar flow passing through the channel has a parabolic profile with the flow velocities being the highest in the center and the slowest at the channel walls (Figure 1a). AF4 has a focusing and an elution step. Sample injection takes place during the focusing step; whereas size-based separation occurs during elution (see Section 2.5). One of the benefits of AF4 is that the separation takes place without the use of stationary phase. This avoids mechanical and shear stress that could cause entanglement and alterations in the native conformation of studied biomaterial.

**Figure 2:**
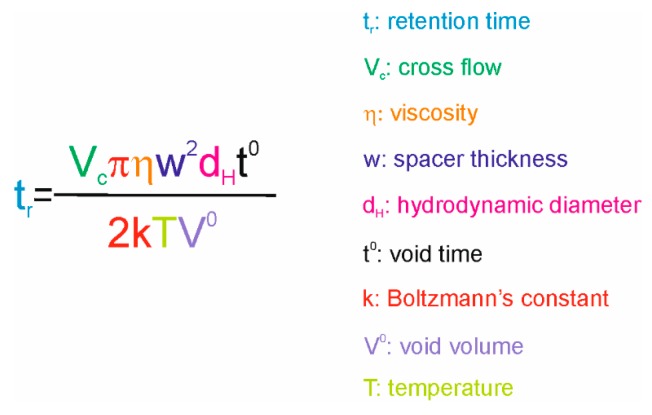
Retention time (t_r_) depends on the diffusion co-efficient of the analyzed sample, which correlates with the hydrodynamic diameter (d_H_). Several parameters affect retention time and have inter-dependent effects. In general, cross flow (V_c_) has the strongest impact on separation. Void time (t_0_) and void volume (V_0_) are dependent on the channel thickness (w), whereas diffusion and eluent viscosity are temperature-dependent. In addition, the eluent, channel geometry, and the channel-flow rate affect retention time.

Separation of sample components is affected the most by the applied external field—the cross-flow. It is perpendicular to the channel flow and pushes the sample components towards the accumulation wall. This force is counteracted by diffusion of molecules away from the wall (Figure 1a). As a result, each sample component equilibrates at a distance from the accumulation wall that depends on the diffusion coefficient (D) and the hydrodynamic diameter (d_H_) of the studied sample [1,2], as well as the applied fractionation conditions (Figure 2). Normal elution mode applies to sample components that are smaller than ~0.5−1 μm in diameter [3,4,5] and are valid for most viruses and virus-like particles (VLP). In normal elution mode, large sample components with small diffusion coefficients equilibrate closer to the accumulation wall than do small particles with large diffusion coefficients. Therefore, smaller particles experience the higher velocity flow in the central region of the channel and elute first (Figure 1a). For particles that have diameters larger than ~1 μm, diffusion away from the accumulation wall is negligible and elution proceeds from larger to smaller sample components. Such reverse elution order (steric elution mode) can take place for rod-shaped particles, if the ratio of the longitudinal axis and the diameter is high [6] and is thus possible for rod-shaped and filamentous viruses. There is no upper molar mass limit in AF4 separation [7]. Detailed description of the AF4 principles, theory, and applications can be found from many available reviews and textbooks [4,7,8,9,10,11,12,13]. Although other sub-techniques of field-flow fractionation, such as centrifugal (or sedimentation) field-flow fractionation or electrical field-flow fractionation show great potential for virus-related studies, here we focus on AF4.

The most common online detectors coupled to AF4 are multi angle light scattering (MALS), dynamic light scattering (DLS), ultraviolet (UV), and refractive index (RI) detectors. However, for instance, fluorescence detectors can provide valuable information on components tagged with fluorescent proteins or labels. Light scattering detectors enable the determination of the radius of gyration (R_g_—MALS), radius of hydration (R_h_—DLS), shape factor (R_g_/R_h_), and polydispersity index. Combination of the data obtained from the concentration (UV, RI) and light scattering detectors enables the collection of data on molecular weight (M_w_) distribution. However, AF4 theory can also be utilized to calculate hydrodynamic diameter (d_H_) based on the retention time (t_r_) obtained with the applied experimental parameters, provided that constant cross flow has been applied and conditions providing high retention are used (see Section 2.4) (Figure 2). If cross-flow gradients are used, d_H_ can be determined by performing AF4 analysis with standards that cover the sizes of studied sample components and making a calibration curve between d_H_ and t_r_. Finally, molecular mass and volume information can be used to calculate the apparent density of the studied sample components.

The field-flow fractionation technique was introduced in 1976 with self-made instrumentation [14]. One of the first studies reported the use of symmetric field-flow fractionation with two accumulation walls to determine the diffusion coefficients of bacteriophages Qβ, f2, MS2, P22, and ϕX174 [1]. The potential of field-flow fractionation for separating, identifying, purifying, quantitating, and characterizing viruses was recognized already then.

**Figure 3:**
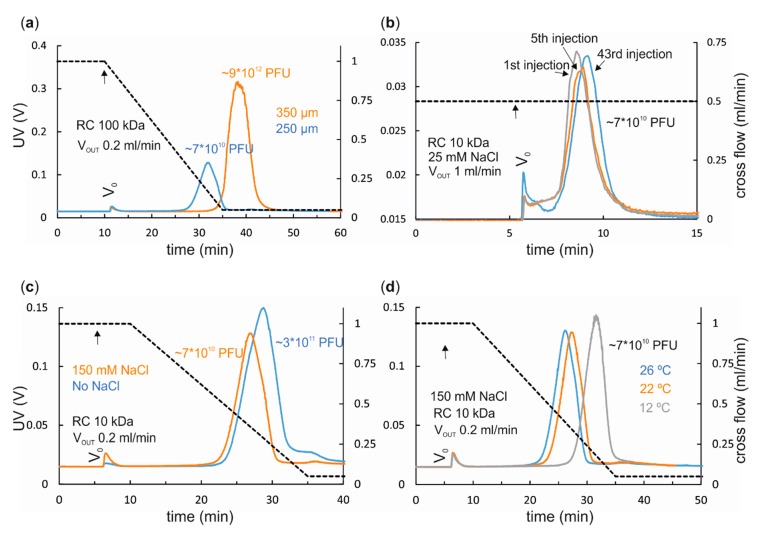
Effects of instrumental parameters on AF4 separation of enveloped bacteriophage ϕ6. (**a**) Thinner spacer advances elution (spacers of 250 and 350 µm in orange and blue, respectively). (**b**) Adsorption of sample components on membrane surface causes membrane fouling (membrane aging) that influences retention time. (**c**) The ionic strength of the eluent affects retention time via its influence on the electrostatic interactions between the sample components, and the sample components and the membrane. (**d**) Decreased channel temperature slows down diffusion and retards elution. Cross-flow gradients are shown with black dashed lines (right y-axis). Transition from focusing to elution is indicated with an arrow. UV detector response is given in volts (V) (left y-axis). Eluent was 20 mM potassium phosphate buffer (pH 7.2), 1 mM MgCl_2_. NaCl concentration of eluent (if present), channel flow rate (V_out_), void peak (V_0_), injected virus amounts (PFU), and membrane MWCO are indicated in the corresponding panels. A spacer with a 250 μm nominal thickness was used in **b**–**d**. The AF4 instrument used was from Postnova and its operation is described in [32]. ϕ6 was pre-purified with polyethylene glycol precipitation and two subsequent ultracentrifugations [34], Appendix A.

**Figure 4:**
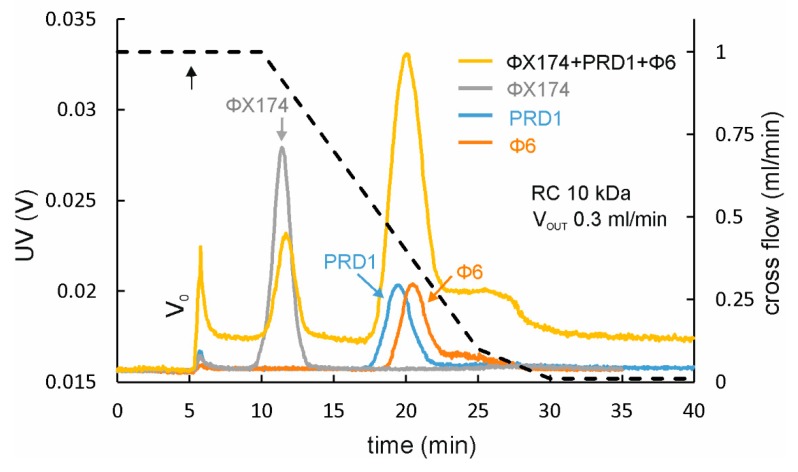
AF4 separation of different-sized spherical bacteriophages. Baseline separation is obtained for ϕX174 (ø ~25 nm; grey line) and PRD1 (ø ~63 nm; blue line) or ϕ6 (ø ~71 nm; orange line) when analyzed separately. However, PRD1 and ϕ6 with similar sizes cannot be separated (peak at ~20 min) when the fractionation is performed for the mixture of ϕX174, PRD1, and ϕ6 (yellow line). The cross-flow gradient is shown with black dashed line (right y-axis). Transition from focusing to elution is indicated with an arrow. UV detector response is given in volts (V) (left y-axis). A spacer with 250 μm nominal thickness was used. Eluent was 20 mM Tris-HCl (pH 7.5), 25 mM NaCl. Injected virus amounts were: ϕX174 ~1 × 10^10^ PFU; PRD1 ~1 × 10^11^ PFU; and ϕ6 ~1 × 10^11^ PFU. An RC membrane with MWCO of 10 kDa and a channel flow rate of 0.3 mL/min was used. V_0_ is the void peak. The AF4 instrument used and its operation is described in [32]. Production and purification of viruses is summarized in Appendix A.

**Figure 5:**
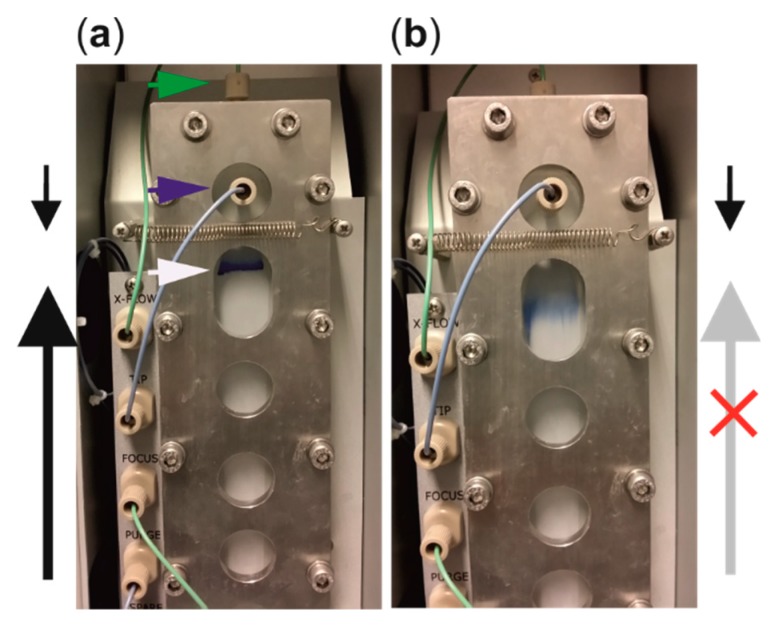
The AF4 experiment is divided into the focusing and elution steps. The pre-stained protein sample was fractionated to visualize (**a**) the focusing site (indicated with a white arrow) and (**b**) the beginning of elution. (**a**) During focusing, sample components concentrate into a thin band (here visible as a blue zone). Focusing is achieved by applying a focusing flow (long black arrow upwards) from an opposite direction in relation to the incoming tip flow that equals to injection flow during the focusing step (short black arrow downwards). (**b**) Switching off the focusing flow (long grey arrow upwards) initiates elution and sample components start moving towards the outlet end of the channel (here downwards), the detectors, and the fraction collector according to their sizes. During elution, the tip flow rate is equal to the sum of channel-flow and cross-flow rates. Ports for the inlet (tip flow in, blue arrow head) and cross flow (green arrow head) in the Postnova analytical channel are indicated.

**Figure 6:**
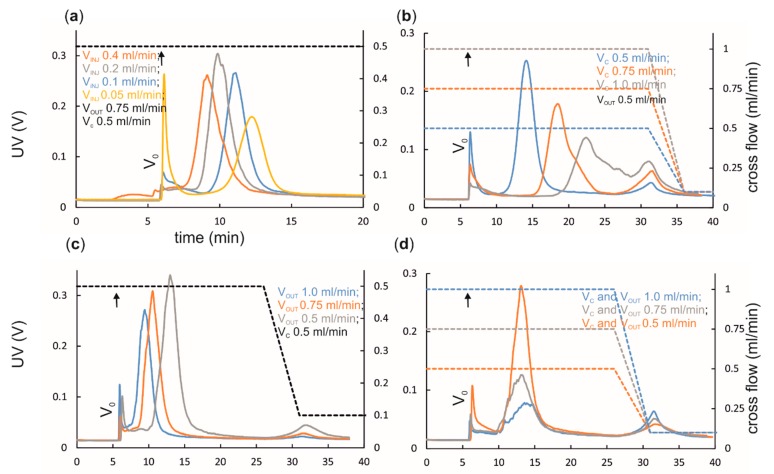
Effect of flow rates on separation and retention time. (**a**) Focusing position (z_foc_) affects retention time. The closer it is to the inlet, the longer is the effective channel length for the separation. Additionally, the retention times increase. (**b**) An increased cross-flow rate (V_c_) promotes equilibration of sample components into a narrower zone closer to the accumulation wall and retards elution. (**c**) Increased channel-flow rate (V_out_) promotes elution and shortens the required analysis time, but may induce dilution. (**d**) Retention time is unaffected if the ratio of cross-flow and channel-flow rate (V_c_/V_out_) is kept constant, whereas resolution and peak intensities can change. Here, increased flow rates induce virus aggregation, observed as an increase in the intensity of the peak eluting at the end of the cross-flow gradient. UV detector response is given in volts (V) (left y-axis). Cross-flow gradients are shown with dashed lines (right y-axis). Transition from focusing to elution is indicated with an arrow. Injection (V_inj_), cross-flow (V_c_), and channel-flow (V_out_) rates are indicated in the figures. V_0_ is the void peak. Spacer with 250 μm nominal thickness was used. Eluent was 20 mM Tris-HCl (pH 7.5), 25 mM NaCl. Injected ϕ6 amount was ~7 × 10^10^ PFU corresponding to ~15 μg [34], see Appendix A.

Virus and VLP preparations purified to near homogeneity are essential to fundamental basic virology but also to several biotechnological, nanotechnological, and medical applications. Large macromolecular complexes such as viruses, VLPs, and their subassemblies, however, are challenging objects to purify. Furthermore, particles that are intended for vaccination or gene therapy need to be pure, stable, homogenous in size, and devoid of aggregates. Same quality requirements also apply for many biochemical and structural studies of viruses and VLPs. The benefits and challenges of AF4 are summarized in Table 1 and discussed below. We summarize the current works where commercial AF4 equipment from Wyatt and Postnova have been applied to study viruses and VLPs. We also provide examples on the effect of experimental parameters on AF4 fractionation of an enveloped bacteriophage ϕ6 with diameter of ~70 nm [15] on a standard analytical AF4 channel using the Postnova instrument (Figure 3, Figure 4, Figure 5 and Figure 6).

## 2. It Depends on Conditions: AF4 Parameters Affecting Separation

AF4 method optimization is done according to the largest sample component that is being analyzed. In general, AF4 theory-based predictions and literature provide a good starting point for methods development. Importantly, the experimental parameters have a complex inter-dependency (Figure 2) and the conditions may need to be re-adjusted several times. However, the obtained results are typically highly repeatable. We have collected the experimental conditions utilized for AF4 studies of VLPs and viruses in Table 2 and Table 3, respectively. Whether AF4 is aimed to be utilized for high-resolution size-separation or as a method to obtain a maximal amount of sample for downstream applications determines the critical points for AF4 method development.

### 2.1. Channel Dimensions

Sample components are separated in a channel. Its shape, length, breadth, and thickness is defined by the used spacer (Figure 1b). Spacer thickness affects separation (Figure 2). Thin spacers shorten the analysis time, and reduce eluent consumption as well as sample dilution due to decreased channel volume [36], whereas thicker spacers provide better separation and may enable higher sample loads [7,13,37]. The effect of 250 and 350 μm spacers on elution of bacteriophage ϕ6 in the standard analytical channel is exemplified in Figure 3a. The use of a thinner spacer promotes virus elution at a higher cross-flow rate, enabling its separation from larger sample components. In general, most virus and VLP works have utilized the 350 μm spacer, but there are also examples where the 250 or 500 μm spacers have been used (Table 2 and Table 3).

Standard analytical channels (~27 cm long, maximal breadth of ~2−2.5 cm; Figure 1b, top) are best suited for analytical purposes due to the limited loading capacity, which is typically below ~0.1 mg. Sample loads can be increased to some extent by using thicker spacers, or by optimizing the focusing site at the point of maximal channel breadth [37] (Figure 1b). The injected sample amount can be increased up to the point where symmetrical elution peaks with unaltered retention times are still obtained. For pre-purified bacteriophage PRD1 sample, the working range using the standard analytical channel and the 350 μm spacer is between ~3 μg and ~3 mg, corresponding to ~10^9^−10^13^ plaque forming units (PFU) [32]. However, when milligram amounts are loaded, the peaks become broadened and skewed, indicating overloading and nonoptimal separation. In general, the applicable loading amount depends on sample concentration, used AF4 parameters, channel dimensions, and sample properties affecting the viscosity of solutions [38], and is typically lower for heterogeneous virus samples compared to purified ones [32,33,34]. Consequently, AF4 purification of viruses directly from cleared cell lysates yields some tens of micrograms per mL of injected lysate [32,34], but the potential yields are obviously highly dependent on the virus and the corresponding production system.

In preparative approaches, the limits of the standard analytical channels can be compensated by making several AF4 fractionations in a row, which is feasible due to the short analysis time (see Figure 3b for example). However, the best option is to use semi-preparative channels with increased maximal breadth at the inlet end (Figure 1b, bottom), while increasing the channel length has little effect on the loading capacity [37]. Semi-preparative channels with a maximum breadth of 5 or 10 cm have been used to separate milligram quantities of solid silica spheres with diameters of 50 and 100 nm [37] and liposomes with mean diameters of ~70 and ~300–400 nm [39].

Miniaturized channels with reduced dimensions enable rapid analysis, resulting in increased sample throughput. In addition, less sample and eluent is consumed [26]. For example, miniaturized ~18 cm long channels enabled the analysis of VLP oligomerization state from one fourth of the sample amount compared to the standard channel [26].

### 2.2. Ultrafiltration Membrane

Ultrafiltration membrane chemistry including its charge, MWCO, hydrophobicity and hydrophilicity, as well as the eluent composition, and the properties of the studied sample components affect the applicable flow rates, recoveries, and retention behavior [7,40,41]. Interactions between the sample components and the membrane are the point of potential failure in AF4. Attractive interactions increase the retention time, whereas repulsive interactions reduce it. However, the surface area for potential adsorption is small and thus the recoveries are generally good. Large rod-shaped sample components have a bigger interactive surface area than small and spherical particles and, thus, they are more prone to interact with the membrane. Regenerated cellulose (RC) with MWCO of 10 kDa is most frequently used for viruses and VLPs, but polyethersulfone (PES) membranes are also occasionally utilized (Table 2 and Table 3). For example, higher recovery of bacteriophage Qβ VLPs was obtained with RC than with PES or cellulose triacetate (TCA) membranes [26]. Accumulation of sample components on the membrane can decrease peak intensity and induce small changes in the retention time. This is often most evident among the first experiments/injections with a new membrane and more evident for low sample loads [42]. The effect of membrane fouling/aging is illustrated in Figure 3b. Sample-membrane interactions can be minimized by first running small amounts of the sample or inert protein, such as bovine serum albumin, to condition the membrane. Depending on the sample, up to ~50 injections can be analyzed with a single membrane.

Membrane MWCO can be selectively chosen to remove sample components that are smaller than the pores of the membrane. For example, sugars and peptides of culture media can be efficiently separated from the viruses by utilizing membrane with MWCO of 100 kDa [32,33,34]. Importantly, membrane MWCO affects the cross-flow rate that can be used, as follows: Higher MWCO allows higher cross-flow rates.

The membrane part that aligns beneath the spacer becomes compressed and intrudes into the channel. Thus, the actual channel thickness is smaller than the nominal value of the spacer. The actual measure for the channel thickness is crucial for accurate hydrodynamic size calculations (Figure 2) and it can be determined experimentally. Importantly, the experimentally determined channel thickness is only valid when the same experimental conditions are used [43]. Furthermore, compression tendency of the membrane depends largely on the backing of the membrane, and it is manufacturer and lot dependent [7,43]. Consequently, a change in the membrane lot can have an impact on the obtained separation and retention time.

### 2.3. Eluent and Channel Temperature

Eluent impacts resolution and yield. One of the benefits of AF4 is the flexibility to adjust the eluent composition, although for viruses and VLPs this is often limited to eluents that keep them biologically intact (Table 2 and Table 3). For therapeutics, some additional limits might arise from the intended applications. The eluent can also be intentionally modified to promote the assembly or disassembly of virions and VLPs during AF4 separation (see below). The chosen eluent needs to be compatible with the selected membrane.

The ionic strength, pH, and the presence of surfactants influence the net charge of the sample components and the membrane. Consequently, either attractive or repulsive sample-sample or sample-membrane interactions can take place. At neutral pH, most ultrafiltration membranes have negative charge [44]. The ionic strength of the eluent affects retention time and peak width. At very low ionic strength, the electronic double layers formed around the sample components are thick [45]. This may advance elution, since sample components equilibrate farther from the accumulation wall and have access to higher flow velocities in the channel (Figure 1a). Increasing the ionic strength of the eluent helps to shield attractive electrostatic forces and can reduce peak broadening that is caused by electrostatic repulsion between the sample components. The effect of increased ionic strength on elution of bacteriophage ϕ6 is exemplified in Figure 3c. Importantly, too high ionic strength results in a very thin electronic double layer, sample components equilibrate close to the accumulation wall, electrostatic repulsion becomes negligible, and hydrophobic and other short-range interactions such as van der Waals forces start to dominate [45]. This potentiates low recovery, high retention, and aggregation. However, AF4 has been successfully used to purify viruses using eluents of high, up to 1.6 M, total ionic strength (see Section 3.1) [33].

Channel temperature influences the separation due to its effects on diffusion and eluent viscosity (Figure 2 and Figure 3d). In some applications, lower temperatures may be preferred to inhibit proteolytic degradation or to maintain virion integrity (e.g., viruses of psychrophiles adapted to low temperatures) during AF4.

### 2.4. Separation Efficiency, Retention Time, Retention Level and Resolution

In AF4, flow conditions are adjusted to result in short retention times, and thus a short total analysis time. Retention level is determined as the ratio of retention time of the peak of the studied specimen (t_r_) and that of the void peak (t_0_). It affects separation, resolution, width of the peaks, and analysis time and is thus an important part of AF4 method development [13,46]. A retention level ≥5 indicates strong retention [7]. The higher the value, the narrower the peaks usually are, the more homogenous the peak content will be, and the more accurate the obtained size estimates are [13]. Retention level can be adjusted by altering instrumental parameters such as flow rates. For example, an increased cross-flow to channel-flow rate increases retention time and, thus, the retention level [13]. Higher retention level can also be achieved by using thicker spacers. For virus-sized particles, adequate retention levels are typically easily achieved, since hydrodynamic diameter positively correlates with the retention level [13]. Retention level values ≥30 are not recommended as the analysis time will be unreasonable long [7,13,46].

For heterologous and complex samples, resolution between the peaks is also important. Resolution between two peaks (R_S_) is calculated from the differences in their retention times divided by the average of peak base widths (R_S_ = Δt/w_b_). Complete resolution is obtained if the R_S_ value is ≥1.5. Resolution can be improved by increasing the cross-flow and channel-flow rates while keeping their ratio constant. This should result in the same retention time but in more narrow peaks [13].

In principle, a single AF4 experiment can separate molecules with a broad size range (~10^3^−10^9^ Da, particle diameter from 2 nm to 0.5−1 μm) [5]. Baseline separation can be achieved with sample components having ~twofold size difference. This is exemplified in Figure 4, where separation of bacteriophages ϕX174 (Ø ~25 nm), PRD1 (Ø ~63 nm), and ϕ6 (Ø ~71 nm) was studied. ϕX174 baseline separates from PRD1 and ϕ6 with the resolution of ~4 for ϕX174 and PRD1, whereas the resolution between PRD1 and ϕ6 is only ~0.3. Consequently, ϕX174 is also well separated from the larger viruses when the viruses were mixed prior to AF4, but the peaks for PRD1 and ϕ6 merge (Figure 4, yellow line). The calculated retention levels for ϕX174, PRD1, and ϕ6 are ~6, ~14, and ~15, respectively, indicating good retention. Importantly, it is possible to observe small size differences with AF4 when purified VLP or virus preparations are compared [21,22,35]. For instance, ϕ6 particles that lack the P3 surface protein are well separated from intact virions [35], even though the size difference in radius is only ~2 nm [47], whereas AF4-MALS studies of purified VLP preparations can detect as small as 1 nm differences in the radius of gyration [21,22]. Obviously, for highly heterogeneous samples, it is challenging to obtain narrow and sharp peaks with base line separation for each sample component population. Sometimes pretreatment of the sample may improve resolution. For example, treatment of culture supernatants by nucleases may improve separation of viruses from the host-derived complexes [32,35].

### 2.5. Optimization of Focusing and Elution Steps: Flow Rates and Time

At the moment there are two commercial suppliers for the AF4 instruments, Wyatt and Postnova. The instruments differ in the number of pumps controlling the flow rates and the positions and numbers of the injection-, focusing-, and purge-flow ports. These differences cause some disparity in the operation of the instruments and programming of the flows during focusing and elution steps. As we are familiar with the Postnova instrument, we focus on the principles for regulating the flow rates therein. Regulation of flow rates in the Wyatt instrument are described in Reference [48].

During focusing, two flows from opposite directions are applied (Figure 5a). In the Postnova instrument, the sample is injected during the focusing step along the injection flow from the tip flow port (Figure 5a, blue arrow head). Typical injection-flow rates range from 0.1 to 0.3 mL/min. Focusing flow is applied from the opposite direction to the injection flow (Figure 5a). The Postnova instrument has a separate port for the focus flow, whereas the focusing flow is applied from the outlet port in the Wyatt instrument. In the Postnova setup the focus-flow rate is determined by the set injection, channel-flow, and cross-flow rates so that V_focus_ = V_c_ + V_out_ − V_inj_. Elution begins by switching off the focusing flow (Figure 5b). During elution, the incoming tip flow rate is the sum of the set channel- and cross-flow rates (V_in_ = V_c_ + V_out_). This applies to the Wyatt instrument as well. The flow rates contribute to the analysis time, retention, resolution, concentration, and yield, and are thus important parameters in AF4. The optimized flow rates are always compromises between the analysis time, dilution, and separation. Notably, as the AF4 method separates virus particles based on the size, flow rates that are optimized for one virus strain are usually easily modified to suit for other virus or VLP particles with similar sizes, but are obviously affected by the biochemical properties of the particle surfaces.

#### 2.5.1. Focusing Step

The AF4 experiment initiates with a focusing step that has a dual role. Firstly, it concentrates the injected sample into a thin zone, preferably less than 5 mm wide, before elution is initiated (Figure 5a, white arrow) [49]. Secondly, sample components are equilibrated at the distance from the accumulation wall, which depends on their diffusion properties at the used experimental conditions, such as cross flow, temperature, and spacer thickness. This is sometimes referred as sample relaxation. Focusing is done by applying two opposing flows, the tip and the focusing flow, from the opposite ends of the channel (Figure 5a). The focusing step is optimized by adjusting the time and flow rates. Sub-optimal focusing results in poor separation and broad peaks, high intensity void peaks (V_0_), and reduced yields (Figure 6a), whereas too long focusing time increases the chance for sample–sample and sample–membrane interactions that promote aggregation and membrane fouling. The focusing time is kept as short as possible.

Sample injection takes place during the focusing step. Thus, the focusing time needs to be long enough to ensure that the sample loop is quantitatively emptied. In practice, the focusing time is typically ~3−5 times longer than it takes to empty one loop volume. Thus, small sample volumes allow short focusing times. However, the injected sample volume has little effect in separation as long as the injected sample amount does not cause overloading [50]. Thus, AF4 is well-suited for the analysis of dilute samples. There are reports where up to 1 L of dilute environmental sample has been injected to AF4 [51]. The drawback in analyzing large sample volumes is the long focusing time.

The position of the focusing site is optimized by modifying the injection and focusing flows (Figure 5a and Figure 6a for an experimental example). Its position affects the effective length of the channel that can be used for separation (Figure 1). When using the Postnova instrument, adjusting the rates of the injection flow (V_inj_) and the cross flow (V_c_) so that their ratio is 0.1 will set the focusing site at ~3.3 cm from the inlet at the site where the breadth of the standard analytical channel is the highest [52]. Spacer thickness has no effect on the focusing site position [52]. Higher V_inj_/V_c_ values result in the focusing site moving closer to the outlet end of the channel, which can impair resolution and decrease retention times (Figure 6a).

#### 2.5.2. Elution Step

During elution, the focus flow is switched off and the sample components are separated according to their hydrodynamic sizes and applied flow rates (Figure 1a and Figure 2). The same cross-flow rate is applied during the focusing step and the beginning of elution. Cross flow has the strongest effect on separation (compare Figure 6b,c). A high cross-flow rate improves separation but also promotes potential sample–sample and sample–membrane interactions, leading to possible aggregation and reduced yields, as illustrated in Figure 6b,d. High cross-flow rates are usually accompanied with high channel-flow rates to obtain good and fast separation [13]. Too low cross-flow rate results in poor resolution, intense void peaks, and broad sample peaks [50]. Due to their large sizes, viruses and VLPs are generally well retained in the channel, and relatively low cross-flow rates, 0.4 − 1.5 mL/min, yield good separation (Table 2 and Table 3 and references therein; Figure 3, Figure 4 and Figure 6). Retention time is unaltered as long as the ratio between the cross flow and the channel flow (V_c_/V_out_) is constant (Figure 6d). Channel thickness affects the optimal cross-flow rate. If the spacer is changed to a thinner one, a higher cross-flow rate has to be applied to obtain same resolution.

Elution can be enhanced by applying cross-flow gradients that can vary from constant to linearly or exponentially decreasing cross-flow rates (see Figure 3 and Figure 6 for examples). Gradients with decreasing cross-flow force result in continuous decrease in retention and shortened analysis time. This option can be used to separate small and large sample components in a single experiment in a reasonable time. For example, linear cross-flow gradients enable the purification of viruses from cleared culture supernatants in ~60 min [32,33,34]. However, cross-flow gradients may have a negative impact on resolution, since sample components of different sizes elute close to each other. In addition, the selectivity in separation is lost when the cross flow reaches zero. Importantly, virus elution at the end of the cross-flow gradient can yield homogenous fractions, if the studied samples are free of aggregates [32,33,34].

Channel flow (V_out_) is kept constant during AF4. It affects the analysis time and separation efficiency, albeit at a lower level than the cross flow (compare Figure 6b,c). However, high flow rates may increase sample dilution (Figure 6c), and thus low channel-flow rates enable better detectability [53]. High flow rates increase the amount of liquid waste generated. For genetically modified or pathogenic viruses and VLPs, generation of large volumes of biohazardous waste can be problematic.

One of the drawbacks of AF4 is the sample dilution that takes place during the separation. In the channel, the sample components accommodate only a narrow (a few micrometers) space above the accumulation wall and most of the channel is occupied with eluent only. However, at the channel outlet, the sample zone becomes mixed with the sample-free eluent and the sample is diluted. Large sample components, such as virus-sized complexes, are more prone to dilution than are small sample components [50]. Dilution can be reduced by modifying the flow rates to result in narrow and high intensity peaks [13,50,53] (Figure 6c) or by utilizing thinner spacers [32]. AF4 purification of bacteriophage PRD1 from culture supernatants using a 250 μm spacer and a standard analytical channel resulted in elution of pure virus in comparable concentration to that of the injected input (dilution factor ~1.5), whereas the use of a 350 μm spacer resulted in ~7-fold dilution [32]. Alternatively, an additional slot pump that removes the sample-free buffer zone can be utilized to obtain more concentrated sample fractions.

## 3. AF4 Applications on Viruses and VLPs

### 3.1. Virus and VLP Production and Purification

Separation of viruses and VLPs from other sample components in a single AF4 experiment provides a real-time monitoring tool for virus and VLP production. Such an application can save time when the production conditions are optimized in terms of maximal yield, quality, or both. Applicability of AF4 to monitor the release of virions from lytic (bacteriophage PRD1) and non-lytic (archaeal virus His1) infections by fractionating culture supernatants has been demonstrated [32,33]. For viruses, such as His1, with slow-growing hosts, the plaque formation may take several days. Thus, the possibility to estimate the relative number of viruses directly from the peak area is an obvious benefit. However, the detection level for the studied viruses was ~10^9^ PFU, which limits the use of this method for viruses producing a low number of particles. AF4 has also been used for determining the optimal production time for polyoma JC virus (JCV) VLPs in insect cells [25].

A single optimized AF4 method has been successfully utilized to purify different viruses directly from cleared culture supernatants of infected cells [32,33,34]. The “one-step” AF4 purification results in a virus specimen that has purity comparable with a specimen that is purified to near homogeneity by three subsequent ultracentrifugations of polyethylene glycol-precipitated viruses. The one-step AF4 purification has been successfully applied for viruses that have variable sizes, morphologies, and surface biochemistries (PRD1, ϕ6, His1, HCIV-1, HRPV-1, and HVTV-1) (Table 3). The collected online MALS data indicated that the virus-containing fractions contained homogenous particle populations with expected sizes [32,33,34]. Importantly, AF4 was successfully used to purify viruses requiring high ionic strength for virion stability using eluents having up to 1.6 M total ionic strength. This is a technical advancement, as such conditions are not compatible with ion exchange chromatography [33]. Since AF4 is based on the hydrodynamic size, separation between mature virions and procapsids having the same hydrodynamic size cannot be obtained [32]. In such cases, additional purification methods that are based on density or mass may be needed. Furthermore, natural variation and heterogeneity in the sizes, e.g., in enveloped and vesicle-like virions, can potentially broaden the peaks in AF4.

### 3.2. AF4-MALS Analysis of Sample Quality and Quantity

AF4 coupled to MALS detectors has been widely used for the quality analysis of the size, size distribution, and presence of fragments, oligomers, and aggregates in purified VLP preparations or to compare the quality of VLPs that derived from different hosts or purification conditions (Table 2 and references therein). AF4-MALS is also applicable to qualitative and quantitative studies of intact viruses (Table 3 and reference therein). The majority of these studies have utilized purified VLP or virus preparations (Table 2 and Table 3). For example, AF4-MALS was used to obtain information on the size and size distribution of polyoma virus VLPs assembled from viral capsid proteins having various affinity tags and on the effect of encapsidated DNA on the assembly and size distribution of VLPs [19,21,22,25]. AF4-MALS has also been used to measure the polydispersity and total virus counts of influenza virus preparations that were derived from different influenza A and B strains [28,29].

VLP aggregation is often associated with a loss of vaccine potency and their presence may introduce additional purification needs that influence the yields and increase production costs. Consequently, the stability of VLPs exposed to storage, pH, freezing and thawing, drying, salinity, or addition of various formulations such as lyophilization excipients has been studied by AF4-MALS using Qβ VLPs [27], hepatitis B virus (HBV) VLPs [17], and murine polyoma virus (MPV) VLPs [23]. In addition, the impact of a production host on HBV VLP aggregation tendency has been studied [17]. The presence of aggregates, their reversible disaggregation to monomeric viruses, and the effect of added excipients and temperature on size distribution of influenza viruses has also been successfully examined by AF4-MALS [28].

### 3.3. Virus Assembly and Disassembly, and Virion Composition

Understanding the principles of assembly and disassembly are fundamental questions of virology and also valuable for obtaining high amounts of premium quality VLPs or virions. VLP and virion assembly relies on the self-assembly properties of subunits and depends on the pH, the presence of certain cations, the ionic strength, and sometimes on the specific host and/or virus proteins. AF4 has been used to study the pH and Ca^2+^ requirements for efficient self-assembly of MPV VLPs [20]. It was also utilized in methods development where the recovery and homogeneity of VLP preparations assembled from purified capsomers was improved ~20% by changing the conventional dialysis method to a simple dilution-based approach promoting self-assembly [24].

In production of VLPs for therapeutic applications, controlled dissociation of VLPs is often used to release impurities encapsidated in the VLPs during particle assembly. By using AF4 and eluent composition preventing reassembly of MPV VLPs, efficient separation of VP1 pentamers that are the building blocks for VLPs was obtained [19]. AF4-MALS was also used to study the effect of NaCl and sodium bicarbonate on dissociation of JCV VLPs to VP1 pentamers [25].

AF4 can also be used to analyze the composition of virions and to produce functional subassemblies. In a recent study, transcriptionally active ϕ6 nucleocapsids were purified by AF4 fractionation using particles which were pre-treated with detergent to remove the viral lipid envelope [35]. In addition, other chemical treatments were performed and their fractionation to soluble sample components and virion subassemblies were studied using AF4 [35]. In another study, Proteinase K treatment of HPRV-1 particles prior to AF4 revealed that the observed heterogeneity in particle sizes determined by AF4-MALS was likely due to differences in the composition of the spike proteins [33] that protrude from the surface of the vesicle-like virus particles [54]. Importantly, AF4 has potential to be used to study the nature of complexes formed between virion components, as illustrated by an AF4 study where dissociation of proteins from the superparamagnetic iron oxide nanoparticles in nonequilibrium AF4 allowed for differentiation between the slowly and rapidly dissociating nanoparticle protein complexes [55].

### 3.4. Other Applications

AF4-MALS has been utilized in an applied environmental virology study, where the effect of viral biomass on the optical backscattering in oceans was studied [31]. For the calculation of refractive indexes, the diameters of four viruses (icosahedral phage MS2, Ø ~25-30 nm; icosahedral tailed phage T4, head Ø ~100 nm; and two new bacteriophage isolates from sea water Y-1, Ø ~50–80 nm, and C-2, Ø ~110 nm) were determined using AF4 and purified virus specimen.

## 4. Conclusions

We have summarized the benefits and challenges of the AF4 technique from the perspective of analyzing and purifying virus-sized macromolecular assemblies (Table 1). One of the key benefits is indisputably the gentle separation principle that avoids mechanical and shear stress. The other benefit arises from the readily adjustable eluent composition that can be tailored to maintain the biological activity of the studied specimen to promote good recovery yields. Alternatively, it can be intentionally modified to induce controlled dissociation of particles. As the size range of AF4 separation ranges from nm to μm size ranges, all viruses and VLPs described to date are applicable to AF4 fractionation. However, the current AF4 research has focused on the study of spherical and tailed viruses only, and the elution properties of long rod-shaped viruses remains to be studied. The possibility to obtain online data on the sample quality is an obvious plus. Although obtaining good separation in AF4 can be tedious, the optimized methods are typically applicable to similarly sized viruses and the analysis are highly repeatable. Analytical and semi-preparative options as well as automated injection and fractionation possibilities provide additional benefits that make AF4 an attractive and versatile tool for studies of viruses and VLPs.

## Figures and Tables

**Table 1:** Advantages and challenges of AF4 in virus and virus-like particle (VLP) applications.

Advantages	Challenges
Gentle non-invasive technique. Automation in injection and fraction collection. Adjustable mobile phase composition. Size-based separation. Short analysis time. Typically high recovery yields. High repeatability between injections. Wide size range for separation in a single AF4 experiment. Easy coupling to various in-line detectors (UV, MALS, DLS, fluorescence). Easy fraction collection for off-line analysis. Various membrane materials available with different MWCO. Membrane-selective AF4 to remove components that are smaller than the membrane MWCO. Up to ~50 injections can be analysed with a single membrane. Slot flow pump option to remove the sample-free eluent. Semi-preparative and mini channels to preparative applications and analysis of scarce samples. Sample pre-processing not required. Relatively cheap running costs.	Tedious optimization. Inter-dependent effects of experimental parameters on retention behaviour. Not routinely available. Potential mixing of normal and steric elution modes if the analyzed sample has high polydispersity in the size range. Potential membrane interactions with sample components. Sample-sample interactions if high sample loads or high cross-flow rate is applied. Lot-to-lot variation in membranes and between manufacturers. Sample dilution during fractionation. Small sample loads in standard analytical AF4. High initial investment costs. Expensive detectors.

**Table 2:** Summary of AF4 studies on VLPs and the AF4 operation conditions used.

Virus ^1)^	Specimen	Analysis/Study	Membrane ^3)^	MWCO, kDa	Spacer, μm	Channel ^4)^	V_C_ Gradient ^5)^	V_C_, Start	V_C_, End	V_OUT_	Total Analysis Time ^6)^ (min)	Eluent	Reference
EV71, CVA16	VLPs (Ø ~30 nm)	Quality^2)^, yield	RC	10	350	S	C	1.0	0	1.2	50 (6 + 30)	50 mM Tris-glycine (pH 7.4), 150 mM NaCl	[16]
HBV (HBsAg)	VLPs (Ø ~23–29 nm)	Quality, salt induced aggregation, stability	RC	10	350	S	C+L	1.0	1.0	1.0	55 (5 + 40 + 5 + 5)	20 mM phosphate buffer (pH 7.4)	[17,18]
MPV	VLPs (VP1) (Ø ~40–60 nm)	VLP characterization: in vitro and in vivo assembled, V_C_ optimization, sample load	RC	10	350	S	C+L	0.75	0	0.75	~60 (10 + 30)	10 mM Tris-HCl (pH 8.0), 50 mM NaCl, 10 mM CaCl_2_ or 20 mM Tris-HCl (pH 7.4), 200 mM NaCl, 1 mM CaCl_2_, 5% (v/v) glycerol	[19]
MPV	Disassembled VLPs, pentamers	Quality	As in [19].									40 mM Tris-HCl (pH 8.0), 200 mM NaCl, 1 mM EDTA, 5 % (v/v) glycerol, 5 mM DTT	[19,20]
MPV	VLPs	Size and size distribution: effect of affinity tags on VP1^7)^ and VP2, effect of packaged DNA or VP2. Method validation^8)^	As in [19].									10 mM Tris (pH 8.0), 50 mM NaCl, 0.01 mM CaCl_2_	[21,22]
MPV	In vitro assembled VLPs (VP1)	Quality	As in [19].									40 mM Tris (pH 7.2 or 8.0), 200 mM NaCl, 5% (v/v) glycerol, 0.05 or 0.2 mM CaCl_2_	[20]
MPV	VLPs	VLP formulation, stability, aggregation, effect of affinity tags (VP1)	As in [19].									PBS (pH 7.4)^9)^	[23]
MPV	In vitro assembled VLPs, VP1 capsomers (wild type and modified)	Formulation of efficient in vitro assembly reaction, VLP quality, recovery	As in [19].									PBS (pH 7.4)	[24]
JCV	VLPs (Ø ~40 nm) and VP1 pentamers	VLP quality, optimal production, effect of packaged DNA, dissociation	RC	10	350	S	C+L	0.75, 1.0, 1.5	0	0.75 or 1.0	50 (10 + 20–35 + 1–5)	VLPs: 20 mM Tris-HCl (pH 7.5), 50/150 mM NaCl. VP1 Pentamers: 280 mM NaCl, 1 mM EGTA, 5 mM DTT	[25]
JCV	VLPs	VLP quality, optimal production conditions	RC	10	440	S	L	1.5	0	0.5	50 (10 + 25)	20 mM Tris-HCl (pH 7.5), 150 mM NaCl	[25]
Qβ, NicQβ	VLPs	Optimization (membranes, flow rates, focusing time). VLP (3.5 MDa) quality & stability. Method validation^8)^	RC (PES, CA)	10	350	S	C+L+L	2.0	0.15, 0	1.5	56 (8 + 18 + 15 + 5)	20 mM Na phosphate buffer (pH 7.0), 150 mM NaCl	[26,27]
Qβ	VLPs	VLP quality	RC	10	350	M	C+L	1.0	0	1.2	31 (6+14+5)	As above	[26]

^1)^ Virus abbreviations: EV71, enterovirus 71; CVA16, coxsackie virus A16; HBsAg, hepatitis B surface antigen; JCV, human polyoma JC virus; MPV, murine polyoma virus, NicQb, VLPs of bacteriophage Qβ that wears covalently linked nicotine haptens. ^2)^ Quality: Molar mass, size, size distribution, heterogeneity, presence of aggregates, oligomers, degradation products. ^3)^ RC, regenerated cellulose; CA, cellulose acetate; PES, polyether sulfone. ^4)^ Channel type: S, standard analytical channel (length ~26 cm); M, mini channel (length ~18 cm). ^5)^ Cross-flow gradient types: C, constant cross-flow rate; L, linear cross-flow gradient. Typical elution programs also utilize rinse periods without applied cross flow at the end of the run (not indicated in the table). ^6)^ Total analysis time is dependent on the time that the analysis is continued after the cross flow has ceased to 0 mL/min. In many of the publications this is not clearly indicated. Focusing and injection time and the length of elution gradient are given in parenthesis. ^7)^ VP, viral protein ^8)^ Method validation: AF4-MALS compared to electrospray differential mobility analysis and transmission electron microscopy (MPV) or size exclusion chromatography and dynamic light scattering (Qβ). ^9)^ PBS, phosphate buffered saline (pH 7.4).

**Table 3:** Summary of AF4 studies on viruses and used operation conditions for standard analytical channel.

Virus ^1)^, Diameter, nm ^2)^	Specimen	Analysis/Study ^3)^	Membrane ^3)^	MWCO, kDa (kDa)	Spacer, μm	V_C_ Gradient ^4)^	V_C_, Start	V_C_, End	V_OUT_	Total Analysis Time ^5)^ (min)	Eluent	Reference
Influenza(~113)	Crude and purified viruses	AF4 method optimization. Polydispersity, effect of purification and excipient addition, virus particle counts. Method validation ^6)^	RC	10	350	L	0.4 or 0.6	0	0.8–1.0	~50–75 (15 + 35–60)	0.1 M K-phosphate (pH 7.4)	[28]
Influenza, AdV	Purified viruses	Aggregation and virus concentration	RC, CA	10	350	L	n.d.*	n.d.	1.0	~25–35	Influenza aqueous, AdV 10% (v/v) glycerol	[29]
Influenza	Purified viruses, culture supernatants	Quantitation and sensitivity, size distribution	PES	10	350	L	0.3	0	1.0	~60 (35)	0.01 M Na-/K-phosphate (pH 7.4), 0.14 M NaCl	[30]
T4, MS2, Y1, C2 (~25–110)	Purified viruses	Effect of viruses on optical backscattering in oceans: refractive index calculations	RC	10	n.d.	C	0.92	0.92	1.9	n.d.	PBS (pH 7.2) or boric acid-buffered saline (pH 9.5), 0.1 % (v/v) Pluronic F68	[31]
PRD1(~63)	Culture supernatants, PEG-precipitate, purified viruses	Virus purification: AF4 method optimization, sample load, quality, progress of infection	RC	100	350, 250	L, L+E	1.0	0.1	0.2, 0.5	~60(5–15 + 25–45)	20 mM K-phosphate (pH 7.2), 1 mM MgCl_2_	[32]
His1, HRPV1, HVTV-1, HCIV-1 (~40–100)	Culture supernatants, PEG-precipitate, purified viruses	Virus purification: yield, quality ^7)^, progress of infection (His1)	RC	100, 10	350, 250	L, L+E	1.0	0.1	0.2, 0.5	~60 (5–15 + 25–45)	20–50 mM Tris-HCl (pH 7.2–7.5), 0.5–1.5 M NaCl, 35–100 mM MgCl_2_, 1–2 mM CaCl_2_, ± 10–28 mM KCl, ±47 mM MgSO_4_	[33]
ϕ6(~71)	Culture supernatants, PEG-precipitate, purified viruses	Virus purification: yield, quality	RC	100, 10	350, 250	L, L+E	1.0	0.1	0.2, 0.5	~60 (5–15 + 25–45)	20 mM K-phosphate (pH 7.2), 1 mM MgCl_2_	[34]
ϕ6	Purified viruses	Virus disassembly, purification of functional subassemblies	RC	10	250	C+L	1.0	0.1	0.2	~50 (5–10 + 5+25)	10 mM K-phosphate (pH 7.2), 1 mM MgCl_2_, ±0.1 mM CaCl_2_, ±150 mM NaCl, ±20 mM EGTA	[35]

* N.d. not determined. ^1)^ Virus name abbreviations: AdV, adenovirus; HVTV-1, Haloarcula vallismortis tailed virus 1; HRPV-1, Halorubrum pleomorphic virus 1; HCIV-1, Haloarcula californiae icosahedral virus 1; T4, MS2, Y1, C2, PRD1, ϕ6 are bacteriophages. ^2)^ D_geo_ = geometric diameter calculated from AF4-MALS derived root mean square radius assuming homogenous or hard spheres or obtained from transmission electron microscopy. For tailed phages, the diameter refers to the capsid (head) size. For pleomorphic viruses the size range of the smallest and largest dimension is given. ^3)^ RC, regenerated cellulose; CA, cellulose acetate; PES, polyether sulfone. ^4)^ Cross-flow gradient type: C, constant cross flow; L, linear cross-flow gradient; E, exponential cross-flow gradient. Typically, elution ends with a rinsing period without applied cross flow. Rinse periods are not indicated in the column. ^5)^ Total analysis time is dependent on the time that the analysis is continued after the cross flow has ceased to 0 mL/min. In many of the publications this is not clearly indicated. Focusing and injection time and the length of elution gradient is given in parenthesis. ^6)^ Method validation: Comparison of AF4-MALS to size-exclusion chromatography coupled to MALS; transmission electron microscopy; atomic force microscopy: median tissue culture dose; fluorescent focus assay; reverse transcription polymerase chain reaction. ^7)^ Quality= molar mass, size, size distribution, purity, heterogeneity, aggregation, and number of infectious viruses.

## Appendix A

**Virus Sample Preparation for AF4**

Bacteriophages ϕ6, PRD1, and ϕX174 were cultured and purified as previously described [32,34] (Table A1). Cell growth was followed by measuring culture turbidity at 550 nm (Chlormic, JP Selecta S.A., Barcelona, Spain). After lysis, cell debris was removed by centrifugation. Viruses were precipitated from the cleared lysate using 10% (w/v) polyethylene glycol (PEG) 6000 and 0.5 M NaCl. Virus precipitates were collected by centrifugation and resuspended in virus specific buffer (~0.01 of the initial volume; see below). The viruses were purified by rate zonal centrifugation in a linear 5–20% (w/v) sucrose gradients prepared in virus-specific buffers. Mature infectious viruses were collected by differential centrifugation and resuspended in virus buffer. Prior to AF4 analysis, aggregated material was removed by centrifugation at 10,000× g for 5 min.

**Table A1:** Summary of virus purification.

	ϕ6	PRD1	ϕX174
Buffer	10 mM K phosphate (pH 7.2), 1 mM MgCl_2_	10 mM K phosphate (pH 7.2), 1 mM MgCl_2_	50 mM Tris-HCl (pH 7.2), 100 mM NaCl
Lysate clearance	~10,000× *g*, 20 min, 10 ºC	~10,000× *g*, 20 min, 10 ºC	~15,000× *g*, 30 min, 10 ºC
PEG precipitation	10% (w/v) PEG, 0.5 M NaCl	10% (w/v) PEG, 0.5 M NaCl	10% (w/v) PEG, 0.5 M NaCl
Collection of PEG precipitate	~10,000× *g*, 20 min, 10 ºC	~10,000× *g*, 20 min, 10 ºC	~15,000× *g*, 30 min, 10 ºC
Rate zonal centrifugation	5–20% (w/v) ^1^, 103,864× g, 50 min, 15 ºC	5–20% (w/v) ^1^, 103,864×g, 55 min, 15 ºC	5–20% (w/v) ^1^, 103,864×g, 75 min, 15 ºC
Differential centrifugation	113,580× *g*, 3 h, 10 ºC	113,580× *g*, 3 h, 5 ºC	144,406× *g*, 2h 20 min, 4 ºC
Given g-values are the max values.			

^1)^ linear sucrose gradient
